# Development of Chitosan/Cyclodextrin Nanospheres for Levofloxacin Ocular Delivery

**DOI:** 10.3390/pharmaceutics13081293

**Published:** 2021-08-19

**Authors:** Federica De Gaetano, Andreana Marino, Alessia Marchetta, Corrado Bongiorno, Roberto Zagami, Maria C. Cristiano, Donatella Paolino, Venerando Pistarà, Cinzia A. Ventura

**Affiliations:** 1 Dipartimento di Scienze Chimiche, Biologiche, Farmaceutiche e Ambientali, Università di Messina, Viale Ferdinando Stagno D’Alcontres 31, I-98166 Messina, Italy; fedegaetano@unime.it (F.D.G.); anmarino@unime.it (A.M.); alemarchetta@unime.it (A.M.); rzagami@unime.it (R.Z.); 2 Istituto di Microelettronica e Microsistemi, Consiglio Nazionale delle Ricerche (CNR-IMM), Strada VIII n. 5-Zona Industriale, I-95121 Catania, Italy; corrado.bongiorno@imm.cnr.it; 3 Dipartimento di Medicina Clinica e Sperimentale, Università “Magna Græcia” di Catanzaro, Viale Europa, I-88100 Catanzaro, Italy; mchiara.cristiano@unicz.it (M.C.C.); paolino@unicz.it (D.P.); 4 Dipartimento di Scienze del Farmaco e della Salute, Università di Catania, Viale Andrea Doria 6, I-95125 Catania, Italy

**Keywords:** levofloxacin, chitosan nanospheres, sulfobutyl-ether-*β*-cyclodextrin, inclusion complex, antibacterial activity

## Abstract

Levofloxacin (LVF) is an antibacterial drug approved for the treatment of ocular infections. However, due to the low ocular bioavailability, high doses are needed, causing bacterial resistance. Polymeric nanospheres (NPs) loading antibiotic drugs represent the most promising approach to eradicate ocular infections and to treat pathogen resistance. In this study, we have developed chitosan NPs based on sulfobutyl-ether-*β*-cyclodextrin (CH/SBE-*β*-CD NPs) for ocular delivery of LVF. CH/SBE-*β*-CD NPs loading LVF were characterized in terms of encapsulation parameters, morphology, and sizes, in comparison to NPs produced without the macrocycle. Nuclear magnetic resonance and UV–vis spectroscopy studies demonstrated that SBE-*β*-CD is able to complex LVF and to influence encapsulation parameters of NPs, producing high encapsulation efficiency and LVF loading. The NPs were homogenous in size, with a hydrodynamic radius between 80 and 170 nm and positive zeta potential (ζ) values. This surface property could promote the interaction of NPs with the negatively charged ocular tissue, increasing their residence time and, consequently, LVF efficacy. In vitro, antibacterial activity against Gram-positive and Gram-negative bacteria showed a double higher activity of CH/SBE-*β*-CD NPs loading LVF compared to the free drug, suggesting that chitosan NPs based on SBE-*β*-CD could be a useful system for the treatment of ocular infections.

## 1. Introduction

**Figure 1:**
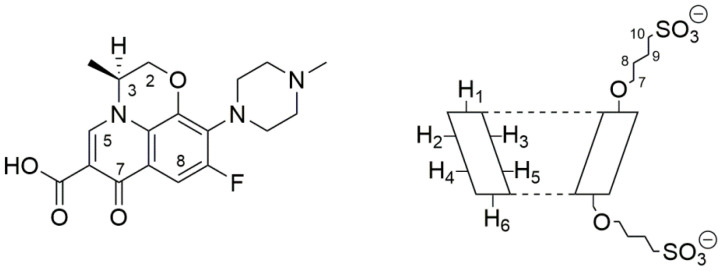
Molecular structure of levofloxacin (LVF) (**left**) and schematic structure of sulfobutyl-ether-*β*-cyclodextrin (SBE-*β*-CD) (**right**).

Levofloxacin (LVF) ((3*S*)-9-fluoro-3-methyl-10-(4-methylpiperazin-1-yl)-7-oxo-2,3-dihydro-7*H*-[1,4]oxazino [2,3,4-*ij*]quinoline-6-carboxylic acid) is an antibacterial chemotherapy belonging to the family of third-generation tricyclic quinolones [1,2], the fluroquinolones. It is the synthetic l-isomer of the racemic ofloxacin, with an atom of fluorine in ninth position (Figure 1).

LVF shows activity widely extended compared to the racemic mixture and the *R*-isomer against positive, negative, and atypical bacteria (aerobic and anaerobic) [3]. It inhibits bacterial topoisomerase IV and DNA gyrase (those two are classified as type II topoisomerases), blocking the transcription and replication of the bacterial DNA [4]. At therapeutic doses, its activity is selective against bacterial topoisomerases, saving human cells. Furthermore, it is well tolerated and excreted largely as an unchanged drug in the urine. LVF has been approved for the treatment of infections involving different organs, such as the respiratory tract, skin, urinary tract, and others, and not least to treat eye infections [5,6].

Both Gram-positive and Gram-negative bacteria are threats of ocular tissues. They can be associated with any types of eye infection, including conjunctivitis, blepharitis, endophthalmitis, keratitis, dacryocystitis, and orbital cellulitis. The common pathogens include *Staphylococcus aureus*, *coagulase-negative Staphylococcus*, *Pseudomonas aeruginosa*, *Streptococcus pneumonia*, *Escherichia coli*, and *Serratia* species. [7]. Topical antibiotics rather than typical oral formulations are prescribed to treat corneal or conjuntival infections based on the assumption that high levels of the drug can be delivered directly to the site of infection, exceeding what is normally achieved in external ocular tissues by oral routes. In this way, the time of infection could be reduced and, also, the risk of developing side effects [8,9]. Several classes of topical antibiotics exhibit efficacy towards ocular infections, among them fluoroquinolones [10]. Ophthalmic application of fluoroquinolones began in the 1990s, when the second-generation fluoroquinolones, such as ciprofloxacin and ofloxacin, were available in topical form. Today, the third-generation (LVF) and fourth-generation (moxifloxacin and gatifloxacin) fluoroquinolones represent the most valuable antibacterial agents for topical use in ophthalmology. Both moxifloxacin and gatifloxacin show a dual mechanism of action, inhibiting both DNA gyrase and topoisomerase IV, providing better coverage against Gram-positive bacteria [11]. In this way, the antibiotic potency against Gram-positive organisms was increased, while the risk of development of bacterial resistance was reduced. However, LVF demonstrated higher antibacterial potency towards Gram-negative micro-organisms. Due to its higher corneal penetration and low development of resistance compared to other molecules of the same class [1], LVF displays higher activity than moxifloxacin against *H. influenzae* and *P. aeruginosa* [12]. Furthermore, Wang et al. [13] demonstrated in a retrospective study covering the period 2010–2013 that, among eight assayed antibiotics belonging to four categories (cephalosporins, quinolones, aminoglycosides, and phenicols), LVF showed to be more active than neomycin (aminoglycosides class) towards strains responsible for internal ocular infections, but not for external infections. Recent studies [14] demonstrated less cytotoxicity for LVF on human corneal keratocytes and epithelial cells than other fluoroquinolones, such as gatifloxacin, moxifloxacin, ciprofloxacin, and ofloxacin.

Despite these advantages, such as other topical dosage forms available in the market for the treatment of ocular infections, LVF solution at 0.5% *w*/*v* results in poor bioavailability due to the rapid precorneal elimination of the drug. To obtain a therapeutic response, frequent administration is needed, causing antibiotic resistance [15]. Innovative dosage forms are able to overcome this drawback, increasing the residence time of the drug on the ocular surface.

It has been demonstrated that polymeric nanoparticles are able to improve the efficacy of drugs with different activity [16,17,18]. They also represent the most promising approach to increase the activity of antibiotics in eradicating ocular infections and to treat pathogen resistance [19,20,21,22,23,24]. Chitosan (CH) shows good properties to realize nanospheres for ocular administration, because it is a cationic, biocompatible, and biodegradable polysaccharide that has been shown to have good mucoadhesive properties [25,26,27], antibacterial properties, and intrinsic bioactivity [28,29]. Ionotropic gelation is a suitable method to produce CH nanospheres (CH NPs) in aqueous solution, avoiding the use of organic solvents. Different polyanions can be used for CH gelation, such as tripolyphosphate (TPP) or sodium sulfate [30], and, recently, negatively charged CDs, such as caboxymethyl-*β*-cyclodextrin (CM-*β*-CD) and sulfobutyl-ether-*β*-cyclodextrin (SBE-*β*-CD) [31,32]. In this case, macrocycles can be used not only as gelling polyanion for CH, but also as complexing agent for a lipophilic drug [33,34], permitting its encapsulation into NPs produced via aqueous process. Furthermore, the presence of a CD into NPs matrix could influence the encapsulation efficiency and release profiles of both lipophilic [35,36] or hydrophilic drug [37] from the NPs as a consequence of the drug/CD interaction.

Recently, it has been demonstrated that LVF encapsulated within CH NPs gelled with TPP was quickly released [38]. Ameeduzzafar et al. [39] demonstrated that CH NPs converted in “in situ gel” by means of alginate or hydroxypropyl methylcellulose (HPMC) increased the residence time of LVF on the eye surface and was effective towards ocular affections. However, the authors did not refer to the release rate of LVF from NPs, so we cannot exclude that the drug was quickly released from NPs and retained as a free drug from the gel formed in situ.

To prolong the release time of LVF from CH NPs, CDs could be used. Authors have demonstrated that LVF forms a 1:1 inclusion complex with native *β*-CD [40] and hydroxypropyl-*β*-CD [41], increasing its water solubility and prolonging its release in the dialysis experiments with respect to the free drug.

In this paper, LVF-loaded CH NPs based on SBE-*β*-CD (Figure 1) (LVF–CH/SBE-*β*-CD NPs) were prepared for ocular disease treatment. The NPs were characterized for morphology, size, encapsulation parameters, and release rate of LVF in comparison with CH NPs obtained by ionotropic gelation with TPP (LVF–CH/TPP NPs). The complexation of LVF with SBE-*β*-CD was studied in solution by means of NMR and UV–vis spectroscopy. Finally, in vitro studies were performed on *Pseudomonas aeruginosa*, *Escherichia coli*, and *Staphylococcus aureus* to evaluate antibacterial activity of the formulations in comparison with the free drug.

## 2. Materials and Methods

### 2.1. Materials

Levofloxacin hydrochloride (LVF, C_18_H_20_FN_3_O_4_, MW 397.8), low molecular weight chitosan (CH; (C_12_H_24_N_2_O_9_)_n_; 75–85% deacetylated; MW, 50.000–190.000 Da based on viscosity; viscosity: 20–300 cps, 1% solution in 1% acetic acid), sodium tripolyphosphate (TPP), trehalose, and lysozyme (EC 3.2.1.17) were purchased from Sigma-Aldrich Chemie^®^ (St. Louis, MO, USA). Sulfobutyl-ether-*β*-cyclodextrin (SBE-*β*-CD, CAPTISOL^®^, average MW 2162, seven is the average degree of sulfobutyl substitution) was a free sample given by CyDex Pharmaceutical (Lenexa, KS, USA). Double-distilled water filtered through 0.22 μm Millipore^®^ GSWP filters (Bedford, MA, USA) was used throughout the study. All other products and reagents were of analytical grade.

### 2.2. Stability of LVF in Solution

Stability of LVF in aqueous solution was evaluated at pH 5 and in phosphate buffer solution (PBS, pH 7.4). Samples of LVF alone (3 × 10^−7^ M) or in the presence of SBE-*β*-CD (drug: SBE-*β*-CD molar ratio, 1:1) were stored at 25.0 ± 0.1 °C and 37.0 ± 0.1 °C in the dark for 7 days. At fixed time intervals, the solutions were analyzed by HPLC (Prominence LC-20AB, Schimadzu, Italia) on an RP-C18 column (100 mm × 4.6 mm I.D., 5 µm particle size) and eluted isocratically with a mixture of triethylamine 1% (*v*/*v*, pH 3.3 adjusted with orthophosphoric acid) and acetonitrile (76/24, *v*/*v*). The flow rate was fixed at 1 mL min^–1^ and the UV detection wavelength used was 290 nm.

### 2.3. Evaluation of LVF/SBE-β-CD Interaction in Solution

Aqueous solutions at pH 5 or PBS (pH 7.4) containing a fixed amount of LVF (3 × 10^−7^ M) were added to an increasing amount of SBE-*β*-CD (1:1, 1:2, 1:5, 1:10 and 1:50 LVF/SBE-*β*-CD molar ratio). The solutions were stirred at 25.0 ± 0.1 °C in the dark for 5 h, and then analyzed by UV–vis spectroscopy in the 200-400 nm spectral range. UV–vis spectra were performed using a FullTech Instruments (Roma, Italy) double-beam spectrophotometer mod PG T80 (resolution 0.001 × 10^−3^ absorbance units; signal-to-noise ratio, 1 × 10^−4^), employing one centimeter rectangular quartz cells (Hellma, Milano, Italy).

Samples of LVF and SBE-*β*-CD, in equivalent concentrations (10 mM), and LVF/SBE-*β*-CD inclusion complex (1:1 molar ratio), prepared in D_2_O, were transferred to 5 mm NMR tubes for spectra acquisition. A Varian Unity Inova 500 MHz (11.75 T), operating at 300 K, was used as the instrument. To avoid the addition of external reference, which could interact with SBE-*β*-CD, the residual water peak (4.67 ppm) was used as the internal ones. Diffusion ordered spectroscopy (DOSY) spectra were recorded with the Dgc-steSL_cc (DOSY gradient compensated stimulated echo with spinlock and convection 160 compensation) HR-DOSY sequence. The pulsed gradient range amplitudes were 0.1067–0.5334 T/m at a diffusion time of 0.06 s. The processing program (DOSY macro in the Varian instrument) was run, with the data transformed using fn = 32 K and lb = 0.3. Rotating frame Overhauser effect spectroscopy (ROESY) spectra were recorded using the ROESYAD sequence (transverse cross-relaxation experiment in rotating frame with adiabatic mixing pulses), with a mixing time of 500 ms.

### 2.4. Preparation of the NPs

Empty CH NPs were obtained by ionotropic gelation method using SBE-*β*-CD or TPP as a gelling agent. Briefly, the CH solutions were prepared by dissolving the polycation (10 mg) in 1% acetic acid solution (5 mL), previously adjusted to pH 5 with the addition of NaOH 2 M drops. These solutions were stirred for 24 h to allow a better solubilization of CH, then 1.5 mL of SBE-*β*-CD (5 mg/mL) or 1.5 mL of TPP (3 mg/mL) in aqueous solution at pH 5 were added dropwise to the polycation solutions under continuous magnetic stirring. The obtained NPs were maintained under stirring for 30 min at room temperature, and subsequently purified by centrifugation at 13,000 rpm for 30 min (ThermoFisher scientific, Heraeus megafuge 16). The pellet was collected and the supernatant centrifuged again two times. The pellets obtained from subsequent centrifugations were reunited, and then resuspended in 1.5 mL of water containing trehalose (5% *w*/*v*) as a cryoprotectant agent and freeze-dried for 72 h (VirtTis Benchtop K Instrument, SP Scientific, Gardiner, MT, USA). The supernatant was discarded.

To obtain LVF-loaded CH/SBE-*β*-CD NPs (LVF–CH/SBE-*β*-CD NPs) and LVF-loaded CH/TPP NPs (LVF–CH/TPP NPs), different amounts of the drug (2 mg and 3 mg) were added to polyanion solution or to CH solution, respectively. The same procedure previously described was performed and the supernatant was used for indirect measurement of LVF encapsulated into the NPs.

### 2.5. Characterization of Chitosan NPs

The freeze-dried empty NPs and NPs loading LVF were collected and weighed to determine the yield %, following the formula:

Yield % = (Effective yield/Theoretical yield) × 100

The encapsulation efficiency (E.E.) and the drug content (D.C.) percentage of LVF-loaded NPs were determined by quantification of the drug in the supernatant. Before the analysis, the supernatant was filtered (LLG-Syringe filters Spheros, Meckenheim, Germany, 0.45 μm Ø 25 mm), then the amount of LVF was estimated by UV–vis spectroscopy in the 200–400 nm spectral range by using the same spectrophotometer previously reported. All measurements were carried out at 25.0 ± 0.1 °C and run at least three times. The E.E. (%) and the D.C. (%) were calculated according to the following equations:

E.E. (%) = [(Theoretical LVF in mg − LVF in supernatant in mg)/Theoretical LVF in mg] × 100

D.C. (%) = [(Theoretical LVF in mg − LVF in supernatant in mg)/Weight of recovered NPs] × 100

The mean hydrodynamic radius of redispersed lyophilized NPs was determined by photon correlation spectroscopy (PCS) using a Zetasizer Nano ZS (Malvern Instrument, Malvern, U.K.). The samples were analyzed at 25 ± 1 °C at a 173° angle with respect to the incident beam. The deconvolution of the measured correlation curve to an intensity size distribution was achieved by using a non-negative least-squares algorithm. The surface charge (ζ-potential values) of all samples was measured by using a Zetasizer Nano ZS (Malvern Instrument, Malvern, UK). A He−Ne laser at a power P = 4.0 mW and λ = 632.8 nm was used. The results are reported as the mean of three separate measurements on three different batches ± the standard deviation (S.D.).

Morphological characterization was carried out using a Jeol JEM 2010F (JEOL Ltd. Tokyo, Japan) transmission electron microscope (TEM) at 200 kV accelerating voltage.

### 2.6. Degradation of NPs in the Presence of Lysozyme

Freeze-dried LVF–CH/SBE-*β*-CD NPs and LVF–CH/TPP NPs (10 mg for each sample) were resuspended in 2 mL of PBS (pH 7.4), then added to 1 mL lysozyme solution (1 mg/mL) and maintained at 25.0 ± 0.1 °C. The suspensions were analyzed at different times (0, 30, 60, 180, 300 min and 24, 72, 96 h) to evaluate changes in size and ζ using the apparatus previously described.

### 2.7. In Vitro LVF Release from NPs

The in vitro release of LVF from NPs was evaluated by using dynamic Franz diffusion cells (LGA, Berkeley, CA, USA). These systems were composed of a donor and a receptor compartment, a diffusional surface area of 0.75 cm^2^, and a volume of 4.75 mL of the receptor compartment. For in vitro release studies, a synthetic cellulose membrane (molecular cut-off 8.000 Da) was placed between the two compartments. Free LVF (1.25 mg) was solubilized in 500 μL of PBS (pH 7.4); CH/SBE-*β*-CD NPs and CH/TPP NPs loading the same amount of the drug were resuspended in 500 μL of PBS (pH 7.4). An aliquot of 200 μL of each sample was placed in the donor chamber. The receptor compartment was filled with PBS (pH 7.4) and was maintained at 35.0 ± 0.1 °C under continuous stirring [42]. At fixed times (1, 3, 5, 24, 48 and 72 h), 500 μL of each sample was collected from the receptor compartment and analyzed by HPLC to quantify LVF using the same operative condition previously described (see Section 2.2). The collected volume was replaced by fresh PBS (pH 7.4). The experiments were conducted in triplicate and the results were expressed as mean ± S.D. The data obtained from in vitro release studies were analyzed by using different equation models to determine the kinetics of release. Thus, diagrams were constructed by plotting the cumulative percentage of released drug vs. time (zero-order model), log cumulative percentage of remaining drug vs. time (first-order model), and cumulative percentage of released drug vs. square root of time (Higuchi models) [43].

### 2.8. Strains

The following strains were used in this study: *Pseudomonas aeruginosa* ATCC 9027, *Escherichia coli* ATCC 10536, and *Staphylococcus aureus* ATCC 6538.

### 2.9. Minimum Inhibitory Concentration (MIC) and Minimum Bactericidal Concentration (MBC) Evaluation

The bacteriostatic and bactericidal properties of LVF–CH/SBE-*β*-CD NPs and LVF–CH/TPP NPs were evaluated in comparison with free LVF, free CH, and empty NPs. The antimicrobial activity was evaluated by determining the minimum inhibitory concentration (MIC) and the minimum bactericidal concentration (MBC) values against the microbial strains described above. The MIC was assessed by broth microdilution method in accordance with EUCAST standardized methods [44]. Briefly, a suspension in growth medium (Müeller–Hinton broth (MHB, Oxoid, Milan, Italy)) was prepared for each bacterial strain, with an optical density equal to 0.5 McFarland (1.5 × 10^8^ CFU/mL). After obtaining a concentration of 10^5^ CFU/mL using appropriate dilutions, each suspension was inoculated in a 96-well microtiter plate containing a serial 2-fold dilution of LVF–CH/SBE-*β*-CD NPs, LVF–CH/TPP NPs, free LVF, free CH, and empty NPs. MIC values, corresponding to the lowest concentration exhibiting no visible bacterial growth, were read after 24 h of incubation at 37 ± 1 °C. The MBC was determined by plating 10 μL from each well showing no turbidity onto Müeller–Hinton agar plates. After incubation at proper conditions, MBC was read as the lowest concentration able to kill 99.9% of the initial inoculum.

### 2.10. Statistical Analysis

One-way ANOVA testing was carried out to determine statistical significance. A Bonferroni *t*-test was used to validate the ANOVA test, and *p* < 0.05 was considered as statistically significant.

## 3. Results

Before starting the study, we evaluated the stability of LVF at the pH value used for the preparation of NPs (pH 5) and in the medium used for in vitro release studies (PBS, pH 7.4), in the absence and presence of SBE-*β*-CD. No significant degradation of LVF was observed at the experimental conditions, as evidenced by HPLC analysis for the free drug and complexed with SBE-*β*-CD (data not shown).

### 3.1. Study of LVF/SBE-β-CD Interaction

**Figure 2:**
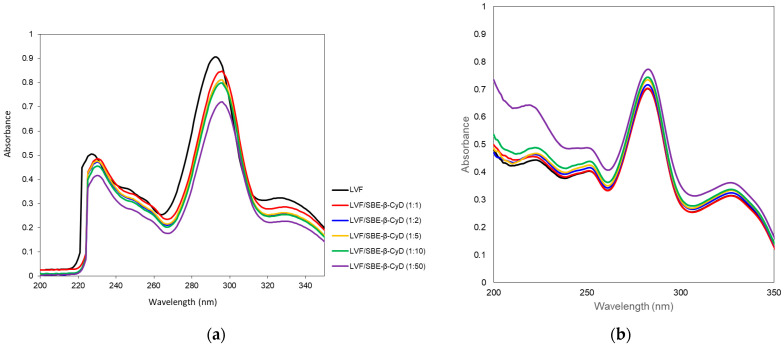
UV–vis spectra of LVF alone and in the presence of an increasing amount of SBE-*β*-CD at pH 5 (**a**) and in PBS pH 7.4 (**b**).

The interaction of LVF with SBE-*β*-CD was investigated by UV–vis and ^1^H-NMR spectroscopy. LVF undergoes acid–base equilibria to form ionic, neutral or zwitterionic species as a function of the pH [45]. Changes in the pH value shift the equilibrium between the different forms, influencing the magnitude of LVF/SBE-*β*-CD interaction. For this reason, UV–vis spectra of LVF alone and in the presence of increasing amounts of SBE-*β*-CD (1:1, 1:2, 1:5, 1:10 and 1:50 molar ratio) were performed in aqueous solution at pH 5 (Figure 2a) to mimic the operative parameters in which NPs were prepared, and in PBS (pH 7.4) (Figure 2b), which is the medium used for the release studies. LVF shows two UV bands in the range 280–310 nm and 320–335 nm, both due to π → π* transition of the chromophore electrons of the drug. In particular, at acid pH, the first band was centered at 292 nm and the second one at 326 nm. The increase in SBE-*β*-CD concentrations produced a bathochromic effect for both bands of about 3 nm, and a significant reduction in their intensity. These phenomena are the result of the variation of local polarity produced by the inclusion of the chromophore electrons within the hydrophobic CD cavity. This is generally accompanied by the establishment of dipole–dipole, electrostatic, van der Walls and/or hydrogen-bond-type interactions. At pH 7.4, the spectrum of LVF alone showed the first band centered at 282 nm and the second one at 327 nm. The shift of λ_max_ with respect to the acid conditions was due to the prevalence of neutral/zwitterionic species at a pH near the LVF isoelectric point (6.77) [46]. In the presence of increasing SBE-*β*-CD concentrations, the influence on the bands was the opposite with respect to pH 5. A progressive increase in the intensity of the two bands was observed without any shift. Furthermore, the variation of the intensity of the two bands was less than that observed at pH 5, probably evidencing the existence in solution of an interaction between LVF and SBE-*β*-CD, which probably involves different binding processes compared to acid pH.

**Figure 3:**
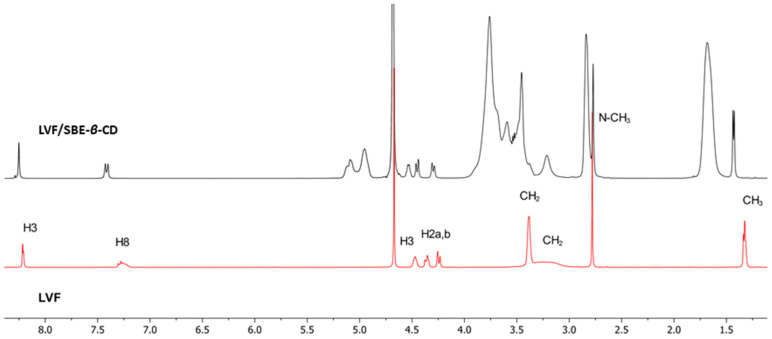
^1^H NMR spectra of LVF/SBE-*β*-CD inclusion complexes (in black) and LVF (in red).

^1^H-NMR spectroscopy confirmed the LVF/SBE-ß-CD interaction. During CDs complexation, chemical and electronic environments of the nucleus are affected, consequently causing the shifts of their corresponding groups (chemical-induced shift, CIS); therefore, ^1^H-NMR spectroscopy is a useful technique employed to investigate the interaction between ligand and carrier molecule. Since the SBE-ß-CD derivative can be considered as a statistical mixture of different stereoisomers, the chemical shifts of its H3 and H5 protons have been assigned by 2D COSY spectrum, as previously reported by some authors [36]. So, the formation of the inclusion complex of LVF in the SBE-*β*-CD cavity was deduced from the chemical shift of the LVF protons in comparison with those of the same protons in the free compounds. In Figure 1, the structure of LVF and schematic representation of SBE-*β*-CD are shown. The ^1^H NMR spectra of LVF and the LVF/SBE-*β*-CD inclusion complex are reported in Figure 3, and the chemical shifts tabulated in Table 1.

The chemical shift changes of the LVF H2, H3, H5, and H8 protons are diagnostics. Most of the variations of LVF chemical shifts were at δ 4–9 ppm, which was distinct and more evident compared to those of the SBE-*β*-CD proton (mainly at δ 3–4 ppm). In the LVF/SBE-*β*-CD inclusion complex, the CIF of H3 LVF proton decreases from 4.47 to 4.54 ppm with the upfield shift of Δδ 0.07. Similarly, H5 and H8 LVF protons showed upfield shifts of Δδ 0.19 (from 7.28 to 7.48 ppm) and Δδ 0.08 (from 8.22 to 8.30 ppm), respectively, after complexation. Furthermore, the CH_3_ protons of 1,4-oxazine ring (position 3 of LVF, Figure 1), decrease from 1.32 to 1.41 ppm with the Δδ 0.09 upfield shift. For the piperazine CH_2_ protons of LVF (Figure 1), the CIF changes are opposed to those of the aromatic ring system, indicating that this ring is outside the SBE-*β*-CD cavity.

**Figure 4:**
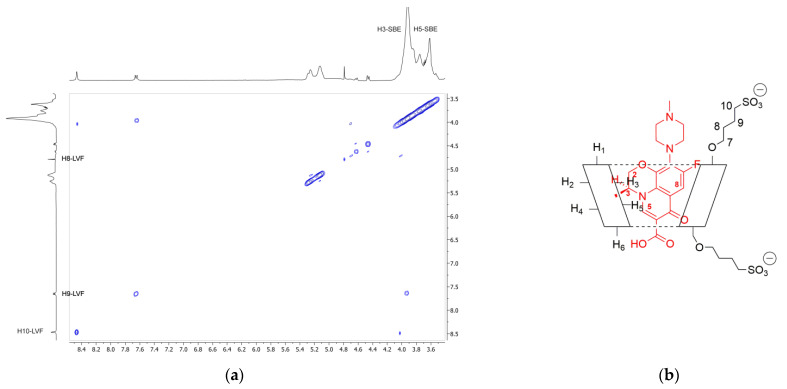
(**a**) Expansion of 2D ROESY plot of LVF/SBE-*β*-CD inclusion complexes with cross-peaks of interest; (**b**) geometry of the inclusion complex deduced form the observed interactions.

In order to confirm the geometry of the LVF/SBE-*β*-CD inclusion complex, rotating frame Overhauser effect spectroscopy (ROESY) experiments were carried out. These types of experiment are suitable to obtain information about inter- and intramolecular interactions between atoms of the host and guest molecules; thus, if two protons from different compounds are in the spatial vicinity within 3–5 Å, an NOE cross-peak is observed in 2D ROESY spectrum. The ROESY spectrum of LVF/SBE-*β*-CD inclusion complex (Figure 4a) shows diagnostic intramolecular NOE cross-peaks for H3 and H5 LVF protons and the internal H3 SBE-*β*-CD protons. Moreover, the internal H5 SBE-*β*-CD protons show a cross-peak with H8 LVF protons. Even the H8 and H9 protons of SBE chains show a weak intramolecular NOE with the methylene protons of LVF 1,4-piperazine ring, whereas the *N*-methyl group shows a contact with the methylene protons of the sulfobutyl chain. These results indicate that the LVF molecule penetrates into the CD from the secondary rim with the aromatic ring system, whereas the methyl-piperazine ring is positioned out of the edge of the cavity. Thus, in accordance with these data, the proposed inclusion complex geometry of LVF/SBE-*β*-CD is reported in Figure 4b.

**Figure 5:**
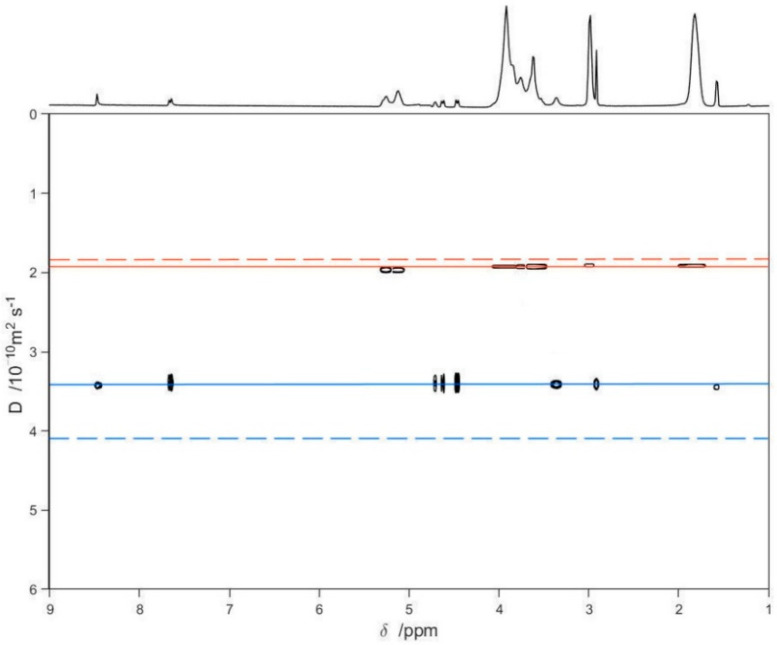
2D DOSY-NMR measurements on the 10 mM D_2_O solutions of LVF, SBE-*β*-CD, and LVF/SBE-*β*-CD 1:1 inclusion complex. The horizontal axis represents the chemical shifts, whereas the vertical axis the diffusion coefficients; the black spots are the resonances of the aqueous solution of the inclusion complex spread in the second dimension according to their measured diffusion coefficient.

Finally, a series of DOSY experiments [36,47,48,49] on free LVF, free SBE-*β*-CD, and the LVF/SBE-*β*-CD 1:1 complex was performed to confirm the inclusion of LVF into the SBE-*β*-CD cavity (Figure 5). Figure 5 shows the diffusion measured for LVF and SBE-*β*-CD, respectively, as green and purple solid lines, whereas the dashed lines are the diffusion coefficients of the LVF/SBE-*β*-CD complex, both as 10 mM solutions. The measurement of the displacement of the solid lines with respect to the corresponding dashed ones (Figure 5) allows a quantitative estimate of the complex formation constant. These results point out that the diffusion coefficient for the LVF diminished considerably (from 4.13 to 3.45 × 10^−10^m^2^/s), and this is indicative of a strong complexation within the SBE-*β*-CD cavity; analogously, the diffusion coefficient of the SBE-*β*-CD also diminished, but slightly (from 1.96 to 1.92 × 10^−10^ m^2^/s).

### 3.2. Characterization of CH NPs Loading LVF

CH NPs were prepared by means of ionotropic gelation using SBE-*β*-CD or TPP as gelling agents, adding different theoretical amounts of LVF in the polyanion or in the polycation solution, respectively. In Table 2, we reported the encapsulation parameters of all prepared formulations.

We observed high and comparable yield percentages for all formulations. The presence of SBE-*β*-CD in the NPs produced a significant improvement of encapsulation parameters compared to LVF–CH/TPP NPs, particularly at the highest theoretical amount of LVF. This trend was probably due to the macrocycle capability to complex LVF, as demonstrated by solution studies. In this way, LVF was mainly retained within the nanospheres as LVF/SBE-*β*-CD inclusion complex, producing a lesser drug loss compared to the TPP-gelled system.

All formulations showed positive ζ values, demonstrating the presence of the gelling agent (SBE-*β*-CD or TPP) on the core of the NPs and the exposition on the surface of the amino groups of CH not interacting with the polyanion (Table 3). SBE-*β*-CD influenced the size of NPs, producing an increase of about two times the hydrodynamic radius compared to LVF–CH/TPP NPs. The macrocycle has a higher number of negative charges for molecules than TPP; this could increase the number of CH chains involved in the formation of NPs and then the sizes. Furthermore, we must consider that SBE-*β*-CD is a bulkier molecule than TPP therefore, a greater distance between CH chains and higher hydration of CH/SBE-*β*-CD NPs with respect to CH/TPP NPs could occur. In any case, the increases in size did not compromise ocular administration.

Based on the good properties in terms of encapsulation parameters of the NPs prepared, starting from a theoretical amount of 3 mg of LVF, for subsequent studies, only these systems were considered.

CH is digested in vivo by lysozyme [50], which commonly exists in various human tissues and fluids, such as tears [51]; then, we assayed the stability of both formulations in the presence of lysozyme by measuring the variations in size and ζ over time. In Table 4, the obtained results are reported.

A reduction in size during the time of observation was evident for both formulations. The majority of the hydrolysis takes place within the first hours of lysozyme addition to the formulations, after this time it proceeds slowly. Probably, this initial fast degradation demonstrated that lysozyme attaches the CH chains located on the surface of the NPs; therefore, it was faster for the larger NPs and gradually decreased as the size decreased. Degradation occurs faster for LVF–CH/SBE-*β*-CD_3_ NPs compared to LVF–CH/TPP_3_ NPs and, up to 7 h from the beginning of the experiments, the size reduction was accompanied by a loss of system homogeneity. We also observed a fluctuation of ζ values (Table 4) for both formulations that is probably related to the variation of charge density during the NPs digestion.

**Figure 6:**
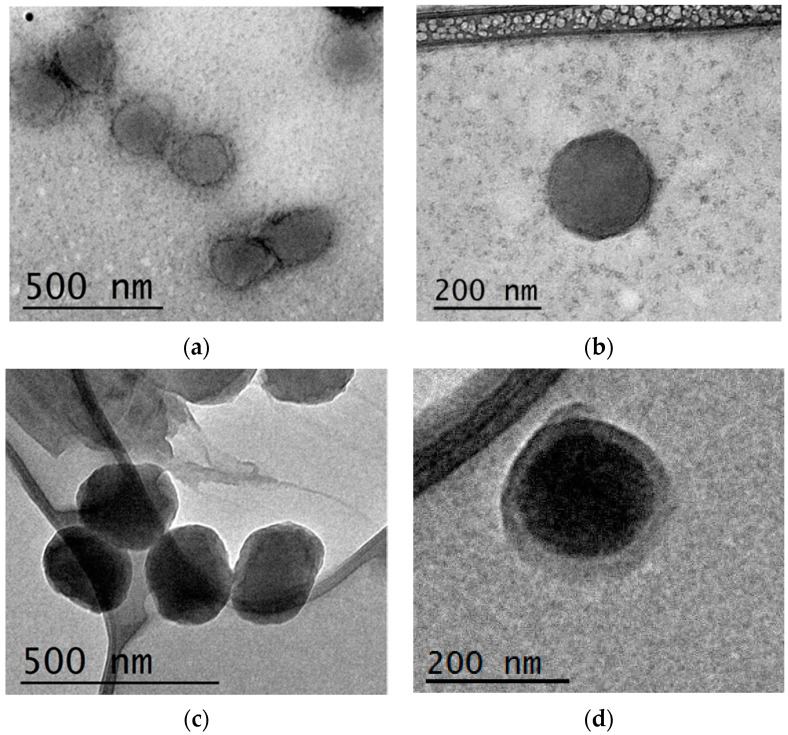
TEM images of LVF–CH/TPP_3_ NPs (**a**,**b**) and LVF–CH/SBE-*β*-CD_3_ NPs (**c**,**d**).

Morphological examination of the NPs was performed with TEM analysis. The obtained pictures, shown in Figure 6, evidenced for both formulations the presence of spherical particles with a high grade of homogeneity. A shell of lesser density compared to the internal core was also observed for LVF–CH/SBE-*β*-CD_3_ NPs due to the presence of non-gelled CH chains on the surface of the NPs [31], and was probably responsible for the faster degradation of these NPs compared to CH/TPP NPs. Lysozyme, in fact, hydrolyses the *β*(1–4) linkages between *N*-acetylglucosamine and glucosamine in CH, and this action could be favored for non-crosslinked CH.

### 3.3. In Vitro Release Profile of LVF-Loaded CH NPs

**Figure 7:**
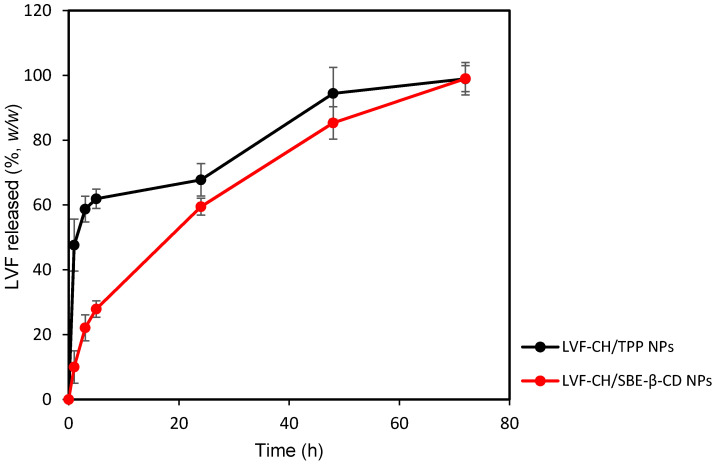
Release profile (%) of LVF–CH/TPP_3_ NPs and LVF–CH/SBE-*β*-CD_3_ NPs, as a function of time. Experiments were carried out using dynamic diffusion Franz cells and a synthetic membrane, and the results are expressed as mean values of three different experiments from three different batches ± standard deviation.

Following physicochemical and morphological evaluation, the ability of LVF–CH/SBE-*β*-CD_3_ NPs and LVF–CH/TPP_3_ NPs to release LVF was evaluated by using dynamic Franz diffusion cells and a synthetic membrane. The release profiles (Figure 7) were biphasic, with a consistent burst effect of about 60% (*w*/*w*) for LVF–CH/TPP_3_ NPs in the first hours of the experiment, while it is reduced to about 20% (*w*/*w*) for LVF–CH/SBE-*β*-CD_3_ NPs. After this time, a sustained release of the remaining LVF in the NPs was observed for 72 h. The burst effect could be principally due to the desorption of LVF superficially adsorbed onto the NPs and/or the rapid diffusion of the drug encapsulated near the surface of the NPs [52]. This external localization of LVF is higher for CH/TPP NPs than CH/SBE-*β*-CD NPs; in fact, in this system, the drug is principally present as an inclusion complex with SBE-*β*-CD, then uniformly accommodates into the matrix. Traces of free LVF are probably present on the surface of the NPs, thus producing a very small burst effect.

The analysis of release data, applying different kinetic models (zero-order, first-order, and Higuchi models), has demonstrated a different release kinetic for the formulations prepared with or without SBE-*β*-CD. LVF–CH/SBE-*β*-CD_3_ NPs showed the best correlation (see R^2^ value in Table 5) by using the Higuchi model, highlighting the release of LVF from the matrix as a square root of a time-dependent process based on Fickian diffusion. None of the above-mentioned models describes the LVF release from LVF–CH/TPP_3_ NPs with any precision, and similar R^2^ values were observed for first-order and Higuchi models, perhaps because different mechanisms could be involved in the release of LVF from this formulation. Furthermore, we observed (Table 5) that, after the burst effect, LVF–CH/SBE-*β*-CD NPs released the drug faster compared to LVF–CH/TPP_3_ NPs, probably due to the higher hydration state of CH/SBE-*β*-CD_3_ NPs, as previously hypothesized.

### 3.4. Antibacterial Activity

Antibacterial activity of free CH, free LVF, empty CH NPs, and LVF-loaded CH-NPs was assayed against *P. aeruginosa*, *E. coli*, and *S. aureus*. The activity of the samples was assayed in terms of minimum inhibitory concentration (MIC) and minimum bactericidal concentration (MBC). Free LVF was active against all tested strains, showing bactericidal activity in the following order: *E. coli* > *S. aureus* > *P. aeruginosa* (Table 6). A limited susceptibility of Gram-negative and Gram-positive strains was observed to free CH also; it exerted its antibacterial activity in the order *E. coli > P. aeruginosa > S. aureus*. The mechanism in which CH acts on bacterial cells is, today, not clearly known; however, it seems that the main mechanism of action of CH is the electrostatic interaction between the positively charged amino group of glucosamine and the negatively charged bacterial wall. In principle, this interaction produces an alteration of the bacterial wall permeability, followed by the leakage of intracellular substances, with a consequent bacterial death. Other mechanisms have been considered, such as the interaction with bacterial DNA, chelating action, and others [53]. The action of CH, leading to cell membrane lysis, has been depicted both in Gram-negative and Gram-positive bacteria [54], even if Gram-negative micro-organisms appear to be more susceptible to CH action. This is probably due to the different components of the cell wall. In the Gram-positive bacteria, a thick peptidoglycan layer containing teichoic acids furnish a negative charge on the surface. Meanwhile, Gram-negative bacteria are characterized by an outer membrane layer containing lipopolysaccharides, which produce a strong negative charge on the surface [55].

Empty CH NPs showed higher antibacterial activity than CH in bulk [54]. This could be due to a deposition of NPs on the bacterial wall, producing a high density of positive charges on the proximity of micro-organisms with respect to CH in bulk, and a consequently a higher destabilizing effect.

As expected, the ability of CH NPs to influence the permeability of the bacterial wall produced a significant increase in LVF activity, probably favoring its penetration within the micro-organisms. In fact, encapsulation of LVF within CH/TPP NPs and CH/SBE-*β*-CD NPs increases its bactericidal activity by about two times. Both formulations showed similar antimicrobial activity, probably related to the same surface properties possessed by the two formulations (similar positive ζ values), and followed the same magnitude order observed for the free drug. However, we must consider that antimicrobial activity was assayed using an amount of the two formulations containing the same amount of drug; then, because NPs prepared with the macrocycle showed higher D.C.% compared to CH/TPP NPs, we can conclude that a lesser dose of LVF–CH/SBE-*β*-CD_3_ NPs can be used to obtain the same effect shown by LVF–CH/TPP_3_ NPs.

## 4. Conclusions

In this work, we have been demonstrated that SBE-*β*-CD is a suitable polyanion to produce CH NPs with good properties for ocular administration of LVF. NPs prepared by ionotropic gelation with the negatively charged macrocycle are characterized in comparison to CH NPs prepared with TPP.

SBE-*β*-CD is able to form a 1:1 inclusion complex with LVF, as demonstrated by UV–vis studies and mono- and two-dimensional NMR spectroscopy. Particularly, ROESY experiments demonstrated the penetration of the LVF aromatic ring system into the CD from the secondary rim, whereas the methyl-piperazine ring is positioned out of the edge of the cavity. As a consequence of the LVF complexation into SBE-*β*-CD, a significant improvement of the technological parameters of NPs was observed, especially in terms of encapsulation efficiency and drug loading. NPs present high positive zeta potential that could ensure their interaction with the negatively charged ocular surface, improving LVF efficacy. LVF-loaded NPs show good antibacterial activity against *E. coli*, *P. aeruginosa*, and *S. aureus*, increasing bactericidal activity by about two times compared to free LVF. Although more in vitro and ex vivo/in vivo studies are required, our results suggest that CH NPs based on SBE-*β*-CD could be a potential delivery system for LVF for the treatment of ocular infections.

## Figures and Tables

**Table 1:** ^1^H NMR chemical shifts in δ and Δδ of LVF protons in free state and LVF/SBE-*β*-CD 1:1 complex (10 mM in D_2_O); for multiplet, doublet, or AB system, the reported δ and Δδ refer to the centered signal.

Protons	LVF	LVF/SBE-*β*-CD	Δδ *
CH_3_	1.32 (d)	1.41	+0.09
*N*-CH_3_	2.78 (s)	2.74	−0.04
(CH_2_)_2_-*N*-CH_3_	3.24 (m)	3.19	−0.05
(CH_2_)_2_-*N*	3.38 (s)	3.43	+0.05
2	4.29 (AB system)	4.36	+0.07
3	4.47 (d)	4.54	+0.07
8	7.28 (m)	7.47	+0.19
5	8.22 (d)	8.30	+0.08

* Δδ = δ_complex_ − δ_free._

**Table 2:** Encapsulation efficiency (E.E.), drug content (D.C.), and yield percentage of CH/SBE-*β*-CD NPs and CH/TPP NPs loading LVF.

NPs Sample *	LVF Theoretical Amount (mg)	Yield (%) ± S.D.	E.E. (%) ± S.D.	D.C. (%) ± S.D.
Empty CH/TPP	-	82.00 ± 4.21	-	-
LVF–CH/TPP_2_	2	82.67 ± 5.49	21.53 ± 1.47	3.47 ± 1.61
LVF–CH/TPP_3_	3	82.5 ± 3.48	25.33 ± 1.24	5.75 ± 1.40
Empty CH/SBE-*β*-CD	-	79.62 ± 7.37	-	-
LVF–CH/SBE-*β*-CD_2_	2	81.05 ± 6.53	41.50 ± 1.19	5.38 ± 1.77
LVF–CH/SBE-*β*-CD_3_	3	81.01 ± 8.51	47.83 ± 2.20	8.65 ± 2.33

* The amount of CH is always 10 mg. The amount of polyanion is 3 mg for TPP and 7.5 mg for SBE-*β*-CD. The number as subscript of NPs acronyms indicates the theoretical amount of LVF in mg (2 mg or 3 mg). LVF was added to CH solutions for the preparation of CH/TPP NPs and to SBE-*β*-CD for the preparation of CH/SBE-*β*-CD NPs. The final volume was maintained at 8 mL for all formulations.

**Table 3:** Hydrodynamic radius (R_H_), polydispersity index (PDI), and zeta potential (ζ) of CH NPs loading LVF.

NPs Sample *	R_H_ ± S.D. (nm)	PDI %	ζ ± S.D. (mV)
Empty CH/TPP	92 ± 43	21	+25.8 ± 3.9
LVF–CH/TPP_2_	85 ± 32	20	+28.0 ± 3.5
LVF–CH/TPP_3_	86 ± 37	20	+26.4 ± 4.6
Empty CH/SBE-*β*-CD	134 ± 41	17	+21.8 ± 5.2
LVF–CH/SBE-*β*-CD_2_	165 ± 49	20	+25.0 ± 2.2
LVF–CH/SBE-*β*-CD_3_	159 ± 52	20	+24.2 ± 4.2

* The amount of CH is always 10 mg. The amount of polyanion is 3 mg for TPP and 7.5 mg for SBE-*β*-CD. The number as subscript of NPs acronyms indicates the theoretical amount of LVF in mg (2 mg or 3 mg). LVF was added to CH solutions for the preparation of CH/TPP NPs and to SBE-*β*-CD for the preparation of CH/SBE-*β*-CD NPs. The final volume was maintained at 8 mL for all formulations.

**Table 4:** Hydrodynamic radius (R_H_) and zeta potential (ζ) of LVF–CH/TPP_3_ NPs and LVF–CH/SBE-*β*-CD_3_ NPs in the presence of lysozyme (1 mg/mL).

NPs Sample	t (h)	R_H_ ± S.D. (nm)	PDI %	ζ ± S.D. (mV)
LVF–CH/TPP_3_ + lysozyme	0	86 ±38	21	+26.1 ± 5.0
	0.5	86 ± 38	21	+25.8 ± 5.2
	1	81 ± 34	20	+26.3 ± 5.3
	3	77 ± 30	21	+26.9 ± 4.5
	5	76 ± 32	20	+26.3 ± 4.4
	7	75 ± 33	20	+25.6 ± 4.5
	24	65 ± 25	20	+25.3 ± 5.0
	48	63 ± 27	21	+24.8 ± 4.1
	72	62 ± 26	19	+23.3 ± 4.8
	96	58 ± 23	20	+24.3 ± 4.6
LVF–CH/SBE-*β*-CD_3_ + lysozyme	0	159 ± 52	16	+23.8 ± 5.3
	0.5	157 ± 64	16	+24.6 ± 5.4
	1	157 ± 70	17	+23.3 ± 4.5
	3	146 ± 58	15	+23.0 ± 5.8
	5	143 ± 57	17	+24.1 ± 5.0
	7	134 ± 56	21	+23.9 ± 4.4
	24	116 ± 48	20	+23.7 ± 4.0
	48	104 ± 43	18	+23.9 ± 4.1
	72	98 ± 43	21	+24.2 ± 3.4
	96	82 ± 29	24	+24.2 ± 4.0

**Table 5:** Regression coefficient (R^2^) and rate constant (K) of LVF release data from LVF–CH/TPP_3_ NPs and LVF–CH/SBE-*β*-CD_3_ NPs according to different kinetic models.

	Zero Order		First Order		Higuchi	
NPs Sample	R^2^	K_0_ (d^−1^)	R^2^	K_0_ (d^−1^)	R^2^	K_0_ (d^−1/2^)
LVF–CH/TPP_3_	0.9247	0.683	0.9498	0.055	0.9408	6.549
LVF–CH/SBE-*β*-CD_3_	0.9458	1.219	0.9276	0.0598	0.997	11.901

**Table 6:** Minimum inhibitory concentration (MIC) and minimum bactericidal concentration (MBC) values (μg/mL) of free LVF, free CH, empty NPs, and LVF-loaded NPs against Gram-negative and Gram-positive bacteria.

Sample	*Pseudomonas aeruginosa*		*Escherichia coli*		*Staphylococcus aureus*	
	MIC	MBC	MIC	MBC	MIC	MBC
LVF	0.5	1	0.016	0.016	0.250	0.250
CH	500	500	250	250	1000	1000
Empty CH/TPP NPs	150	>150	75	>150	150	>150
Empty CH/SBE-β-CD NPs	150	150	18.75	37.5	150	150
LVF–CH/SBE-β-CD_3_ NPs	0.125	0.250	0.004	0.004	0.060	0.060
LVF–CH/TPP_3_ NPs	0.125	0.250	0.004	0.004	0.060	0.060

## Data Availability

The data presented in this study are available on request from the corresponding author. The data are not publicly available due to privacy.
